# Liver fibrosis assessments using FibroScan, virtual-touch tissue quantification, the FIB-4 index, and mac-2 binding protein glycosylation isomer levels compared with pathological findings of liver resection specimens in patients with hepatitis C infection

**DOI:** 10.1186/s12876-020-01459-w

**Published:** 2020-09-25

**Authors:** Naoyuki Ueda, Tomokazu Kawaoka, Michio Imamura, Hiroshi Aikata, Takashi Nakahara, Eisuke Murakami, Masataka Tsuge, Akira Hiramatsu, C. Nelson Hayes, Michiya Yokozaki, Kazuaki Chayama

**Affiliations:** 1 Laboratory Division of Clinical Support, Hiroshima University Hospital, Hiroshima, Japan; 2 Division of Clinical Laboratory Medicine, Hiroshima University Hospital, Hiroshima, Japan; 3 Department of Gastroenterology and Metabolism, Hiroshima University Hospital, 1-2-3 Kasumi, Minami-ku, Hiroshima, 734-8551 Japan; 4 Research Center for Hepatology and Gastroenterology, Hiroshima University, Hiroshima, Japan; 5 RIKEN Center for Integrative Medical Sciences, Yokohama, Japan

**Keywords:** FibroScan, Virtual-touch tissue quantification, Fibrosis index based on four factors, Mac-2 binding protein glycosylation isomer

## Abstract

**Background:**

Evaluation of fibrosis stage is important to monitor progression of liver disease and risk of hepatocellular carcinoma (HCC). While liver biopsy is the gold standard, the method is invasive and faces several limitations. The aim of this study was to determine correlations among METAVIR scores and FibroScan, Virtual-Touch tissue quantification (VTQ), fibrosis index based on four factors (FIB-4 index), and Mac-2 binding protein glycosylation isomer (M2BPGi) level, and for examine differences in the reliability of non-invasive methods to evaluate fibrosis.

**Methods:**

We used liver resection specimens from patients with hepatitis C virus (HCV), correlations were assessed between METAVIR scores and non-invasive method. Receiver operating characteristic (ROC) curves were generated to determine the sensitivity, specificity, and cut off values of the methods.

**Results:**

All Patients group: In F0–2 vs F3–4, the areas under the ROC curve (AUC) (0.85) of FibroScan was significantly higher than that (0.67) of FIB-4 index (*p* = 0.002) and that (0.67) of M2BPGi (*p* = 0.001). The AUC (0.83) of VTQ was significantly higher than that (0.67) of FIB-4 index (*p* = 0.01) and that (0.67) of M2BPGi (*p* = 0.002). In F0–3 vs F4, the AUC (0.86) of VTQ was significantly higher than that (0.65) of FIB-4 index (*p* = 0.04). The AUC (0.89) of FibroScan was significantly higher than that (0.65) of FIB-4 index (*p* = 0.002) and that (0.76) of M2BPGi (*p* = 0.02).

Non-SVR group: In F0–2 vs F3–4, the AUC (0.85) of FibroScan was significantly higher than that (0.84) of FIB-4 index (*p* = 0.02) and that (0.73) of M2BPGi (*p* = 0.003). The AUC (0.84) of VTQ was significantly higher than that (0.74) of FIB-4 index (*p* = 0.04). In F0–3 vs F4, the AUC (0.91) of FibroScan was significantly higher than that (0.67) of FIB-4 index (*p* = 0.003) and that (0.78) of M2BPGi (*p* = 0.02). The AUC (0.88) of VTQ was significantly higher than that of FIB-4 index (0.67) and that of M2BPGi (0.78) (*p* = 0.04).

**Conclusions:**

FibroScan and VTQ best reflected the results of hepatic fibrosis diagnosis using liver resection specimens among the four examination methods evaluated.

## Background

Chronic hepatitis is a disease state most commonly caused by viral infection, in which hepatocytes exhibit inflammation/necrosis, resulting in fibrosis due to repeated regeneration of hepatocytes [1, 2]. Further progression results in liver cirrhosis and an increased risk of developing hepatocellular carcinoma (HCC). The diagnosis of liver fibrosis is very important for determining treatment, predicting chronic liver disease, and assessing the risk of liver cancer [3]. Pathological evaluation of liver biopsy specimens is the gold standard for diagnosis, but because biopsy is invasive, it is difficult to perform frequently [4–7]. In addition, various problems are associated with biopsy that prevent accurate evaluation, such as sampling difficulty, the small amount of specimen obtained, and the subjectivity of the pathologist’s evaluation [8, 9]. For this reason, visual evaluation by ultrasonography and measurements of various blood-based parameters have been conducted as noninvasive alternative methods of hepatic fibrosis diagnosis.

Conventionally, noninvasive methods of assessing liver fibrosis stage include measuring platelet counts, levels of liver fibrosis markers such as hyaluronic acid and type 4 collagen 7S, the aspartate aminotransferase to platelet ratio index, the fibrosis index based on four factors (FIB-4 index), FibroTest, and the serum level of Mac-2 binding protein glycosylation isomer (M2BPGi) [10–14].

In recent years, FibroScan (FibroScan-502®(Echosens, Pari, France)) and Virtual-Touch tissue quantification (VTQ) have gained attention due to advances in ultrasonic diagnostic equipment. Many reports have compared the META-analysis of histological data in VIRal hepatitis (METAVIR) scores of those tests with liver biopsy, both of which are reported to be highly reliable [12, 15–18]. As mentioned previously, however, liver biopsy is invasive and faces limitations with respect to sample amount and pathologist subjectivity. No report has so far compared liver resection specimens in combination with VTQ, FIB-4 index and M2BPGi, and only a few reports have compared liver resection specimens with FibroScan [19].

However, Nagata et al. and Chen et al. reported that FibroScan, VTQ, FIB4-Index and M2BPGi levels are significantly decreased regardless of degree of fibrosis in patients with Sustained Viral Response (SVR) [20–23]. In addition, Bachofner et al. reported that FIB-4 index levels are also lower in patients with SVR after treatment [24].

Therefore, in this study, we compared FibroScan, VTQ, FIB-4 index and the level of M2BPGi with liver fibrosis in both SVR and non-SVR groups.

Correlations were calculated between METAVIR scores and each of these ultrasound tests as well as two hematological marker-based methods: the FIB-4 index and M2BPGi level. ROC curves were generated to determine the sensitivity, specificity, and cutoff values of the tests. From the results, we examined differences in reliability. Inspections were conducted by experienced examiners.

## Methods

### Patients

We recruited 94 adult patients with chronic hepatitis C who underwent surgery for HCC at our hospital between March 2011 and November 2017. Eleven of the patients had been treated by transcatheter arterial chemoembolization as neoadjuvant treatment prior to surgical resection. In addition, patients with liver metastasis were recruited as controls (*n* = 14: frequency of alcohol intake; every day/occasionally/none: 1/7/6). The fibrosis stages of these 14 patients were defined as F0–1 in pathological analyses by liver specimens. Ultrasonography and histology revealed that none of the patients had fatty liver.

Because M2BPGi levels and FIB-4 index levels are known to be significantly decreased regardless of degree of fibrosis in patients with SVR, we compared FibroScan, VTQ, FIB-4 index and the level of M2BPGi with liver fibrosis in both SVR and non-SVR groups.

### Liver stiffness measurements

Liver stiffness was measured using FibroScan and VTQ. FIB-4 index and the level of M2BPGi were measured based on blood parameters. FibroScan measurements should be repeated at least 10 times to obtain a median value and interquartile range. If the rate of successful measurements among the total measurements is < 60% or the interquartile range/median value is > 0.3, the measurements are considered low quality and should not be used in clinical decision making [25]. VTQ was performed using the ACUSON S2000 ultrasound system (Siemens Medical Solutions Inc., CA, USA). Five VTQ measurements were obtained to calculate the average value. FibroScan and VTQ were performed in the intercostal space with the patient lying in the dorsal decubitus position with the right upper limb raised, and liver stiffness measurements were obtained from the right lobe of the liver. FibroScan and VTQ were measured by the same examiner. The M2BPGi level and FIB-4 index were measured in blood samples obtained before surgery. Two ultrasound elastography and blood examinations were performed within 1 month before surgery. HCV was determined by blood test.

### Histological analysis

Liver specimens were obtained by resection of non-tumor tissues at a site away from the tumor becuase non-tumor tissue adjacent to the tumor is largely compressed and cannot be accurately examined. Sites near the tumor could not be evaluated because tumor tissue is broken down by thermocoagulation during liver resection. Similarly, specimens obtained from sites near the resected margins or from the liver surface cannot be evaluated accurately and thus were not included in the study.

Liver resection specimens were fixed in formalin and embedded in paraffin. The sections were subjected to hematoxylin–eosin and azan staining. All surgical specimens were analyzed independently by two experienced pathologists who were blinded to the clinical data. In the case of a discrepancy between the pathologists, the histological grade of each specimen was determined by consensus between them. Fibrosis was staged according to the METAVIR scoring system as follows: F0, no fibrosis; F1, portal fibrosis without septa; F2, portal fibrosis with rare septa; F3, numerous septa without cirrhosis; and F4, cirrhosis [26].

### Statistical analysis

The cutoff value for ROC was taken as the maximum value of [sensitivity + specificity - 1]. SPSS software version 18 (SPSS, Chicago, IL, USA) was used for all statistical analyses. *P*-values less than 0.05 were considered statistically significant. The DeLong method was used to compare AUCs by JMP pro 14.

## Results

### Study population

A total of 108 patients were included in the current study (80 males, 28 females; median age, 69 [21–87] years). The METAVIR fibrosis stage according to FibroScan was F1, F2, F3, and F4 in 2, 36, 39, and 17 of the 94 patients, respectively. The median (range) FIB-4 index in HCC patients was 4.27 (0.3–13.7), and the median (range) M2BPGi level was 2.79 (0.29–8.75) (Table 1). SVR was seen in 30 out of the 108 patients, and failure to achieve sustained virological response (non-SVR) was observed in 64 patients. SVR status was determined by blood test before surgery. Details on non-SVR and SVR are shown in Table 2.

**Table 1:** Clinical and biological characteristics of HCC and liver metastasis patients

	HCC (*n* = 94)	Liver metastasis (*n* = 14)
Age, years^a^	69 (65–87)	60 (21–85)
Gender (male/female)	70/24	10/4
Plt (×10^3^/μl)^a^	14.2 (3.1–34.6)	27.8 (81–195)
BMI (kg/m^2^)^a^	21.5 (18.5–33.5)	21.7 (19.0–27.5)
Alb (g/dl)^a^	4.1 (2.8–5.1)	4.4 (3.4–4.8)
T-Bil (mg/dl)^a^	0.7 (0.3–2.9)	0.8 (0.5–2.1)
AST (U/L)^a^	36.5 (13–114)	19 (14–62)
ALT (U/L)^a^	30.9 (9–114)	16 (10–61)
M2BPGi (COI))^a^	2.79 (0.29–8.75)	0.55 (0.29–3.89)
FIB-4 index^a^	4.27 (0.3–13.7)	2.14 (0.34–1.42)
SVR/non-SVR	30/64	–
Fibrosis (F:0–1/2/3/4)	(2/36/39/17)	(14/0/0/0)
Inflammation (A:0/1/2/3)	(0/17/60/17)	(14/0/0/0)
FibroScan (kPa)^a^	13.9 (2.5–46.4)	5.1 (2.5–8.3)
VTQ (m/s)^a^	1.79 (0.89–3.45)	1.23 (0.96–1.47)
HCC Stage (I/II/III)	(48/35/11)	–

^a^median (range)

*Plt* platelet count, *BMI* Body Mass Index, *Alb* albumin, *T-Bil* total bilirubin, *AST* aspartate aminotransferase, *ALT* alanine aminotransferase, *M2BPGi* Mac-2 binding protein glycosylation isomer, *SVR* sustained viral response, *VTQ* Virtual-Touch tissue quantification, *HCC* hepatocellular carcinoma

**Table 2:** Clinical and biological characteristics of the non-SVR and SVR groups

	non-SVR (*n* = 64)	SVR (*n* = 30)	*P* value
Age, years^a^	69 (21–85)	72 (57–87)	0.60
Gender (male/female)	46/18	23/7	0.12
Plt (×10^3^/μl)^a^	14.2 (3.1–31.0)	12.7 (7.2–34.6)	0.30
BMI (kg/m^2^)^a^	21.5 (18.5–33.5)	22.5 (18.9–30.2)	0.65
Alb (g/dl)^a^	4.1 (2.8–5.1)	4.1 (3.0–4.9)	1.00
T-Bil (mg/dl)^a^	0.7 (0.3–2.9)	0.8 (0.3–2.7)	0.91
AST (U/L)^a^	36.5 (14–114)	33.5 (13–66)	0.07
ALT (U/L)^a^	30.9 (9–114)	25.5 (11–45)	0.08
M2BPGi (COI)^a^	2.79 (0.29–8.75)	2.37 (0.42–8.60)	0.70
FIB-4 index^a^	4.27 (0.34–13.7)	3.48 (1.19–6.33)	0.80
Fibrosis (F:0–1/2/3/4)	(1/21/29/13)	(1/15/10/4)	0.91
Inflammation (A:0/1/2/3)	(0/7/42/15)	(0/5/12/13)	0.24
FibroScan (kPa)^a^	13.9 (2.5–46.4)	11.9 (5.6–46.4)	0.36
VTQ (m/s)^a^	1.69 (0.95–3.28)	1.81 (0.89–3.45)	0.41
HCC Stage (I/II/III)	(30/25/9)	(18/10/2)	0.05

^a^median (range)

*Plt* platelet count, *BMI* Body Mass Index, *Alb* albumin, *T-Bil* total bilirubin, *AST* aspartate aminotransferase, *ALT* alanine aminotransferase, *M2BPGi* Mac-2 binding protein glycosylation isomer, *SVR* sustained viral response, *VTQ* Virtual-Touch tissue quantification, *HCC* hepatocellular carcinoma

### Box plots of FibroScan, VTQ, FIB-4 index and M2BPGi

#### All patients

**Fig. 1:**
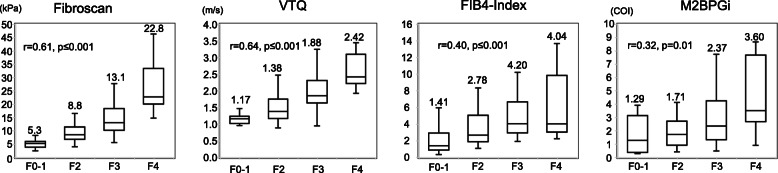
Box plots of the correlations between diagnostic methods and METAVIR fibrosis stages (F0–F4) in patients with liver tumors and hepatitis C infection. FibroScan, VTQ, FIB-4 index, M2BPGi. METAVIR: META-analysis of histological data in VIRal hepatitis scores. VTQ: Virtual-Touch tissue quantification. FIB-4 index: fibrosis index based on four factors. M2BPGi: Mac-2 binding protein glycosylation isomer level

The box plots of the METAVIR scores with respect to each method are shown in Fig. 1. According to Spearman’s rank correlation analysis, positive correlations between each method and the METAVIR fibrosis stage were observed (FibroScan: r = 0.61, *p* ≤ 0.001; VTQ: *r* = 0.64, *p* ≤ 0.001; FIB-4 index: *r* = 0.40, *p* ≤ 0.001; and M2BPGi: *r* = 0.32, *p* = 0.01). The median values for each method were as follows: FibroScan, F0–1: 5.3, F2: 8.8, F3: 13.1, F4: 22.8; VTQ, F0–1: 1.17, F2: 1.38, F3: 1.88, F4: 2.42; FIB-4 index, F0–1: 1.41, F2: 2.78, F3: 4.20, F4: 4.04; M2BPGi, F0–1: 1.29, F2: 1.71, F3: 2.37, F4: 3.60 (Fig. 1). The r values for FibroScan and VTQ were higher than those for FIB-4 index and M2BPGi.

#### Non-SVR group

**Fig. 2:**
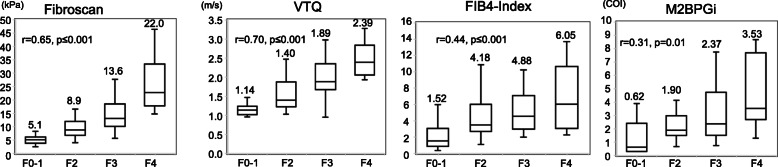
Box plots of the correlations between different diagnostic methods and METAVIR fibrosis stage (F0–F4) in non-SVR patients with liver tumors and hepatitis C. FibroScan, VTQ, FIB-4 index, M2BPGi. METAVIR: META-analysis of histological data in VIRal hepatitis scores. VTQ: Virtual-Touch tissue quantification. FIB-4 index: fibrosis index based on four factors. M2BPGi: Mac-2 binding protein glycosylation isomer level

The results for the non-SVR group are shown in Fig. 2. The fibrosis stages of 14 control patients were defined as F0–1 in the analysis. According to Spearman’s rank correlation analysis, positive correlations between each method and the METAVIR fibrosis stage were observed (FibroScan: *r* = 0.65, *p* ≤ 0.001; VTQ: *r* = 0.70, *p* ≤ 0.001; FIB-4 index: *r* = 0.44, *p* ≤ 0.001; and M2BPGi: *r* = 0.31, *p* = 0.01). The median values for each method were as follows: FibroScan, F0–1: 5.1, F2: 8.9, F3: 13.6, F4: 22.0; VTQ, F0–1: 1.14, F2: 1.40, F3: 1.89, F4: 2.39; FIB-4 index, F0–1: 1.52, F2: 4.18, F3: 4.88, F4: 6.05; M2BPGi, F0–1: 0.62, F2: 1.90, F3: 2.37, F4: 3.53 (Fig. 2). The r values for FibroScan and VTQ were higher than those for FIB-4 index and M2BPGi.

#### SVR group

**Fig. 3:**
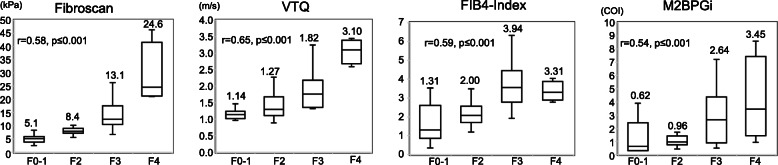
Box plots of the correlations between different diagnostic methods and METAVIR fibrosis stage (F0–F4) in SVR patients with liver tumors and hepatitis C. FibroScan, VTQ, FIB-4 index, M2BPGi. METAVIR: META-analysis of histological data in VIRal hepatitis scores. VTQ: Virtual-Touch tissue quantification. FIB-4 index: fibrosis index based on four factors. M2BPGi: Mac-2 binding protein glycosylation isomer level

The results for the SVR group are shown in Fig. 3. The fibrosis stages of the 14 control patients were defined as F0–1 in the analysis. According to Spearman’s rank correlation analysis, positive correlations between each method and the METAVIR fibrosis stage were observed (FibroScan: *r* = 0.58, *p* ≤ 0.001; VTQ: *r* = 0.65, *p* ≤ 0.001; FIB-4 index: *r* = 0.59, *p* ≤ 0.001; and M2BPGi: *r* = 0.54, *p* ≤ 0.001). The median values for each method were as follows: FibroScan, F0–1: 5.1, F2: 8.4, F3: 13.1, F4: 24.6; VTQ, F0–1: 1.14, F2:1.27, F3: 1.82, F4: 3.10; FIB-4 index, F0–1: 1.31, F2: 2.00, F3: 3.94, F4: 3.31; M2BPGi, F0–1: 0.62, F2: 0.96, F3: 2.64, F4: 3.45 (Fig. 3). The r values for VTQ were higher than those of FibroScan, FIB-4 index and M2BPGi.

### ROC analysis of FibroScan, VTQ, FIB-4 index and M2BPGi

#### All patients

**Fig. 4:**
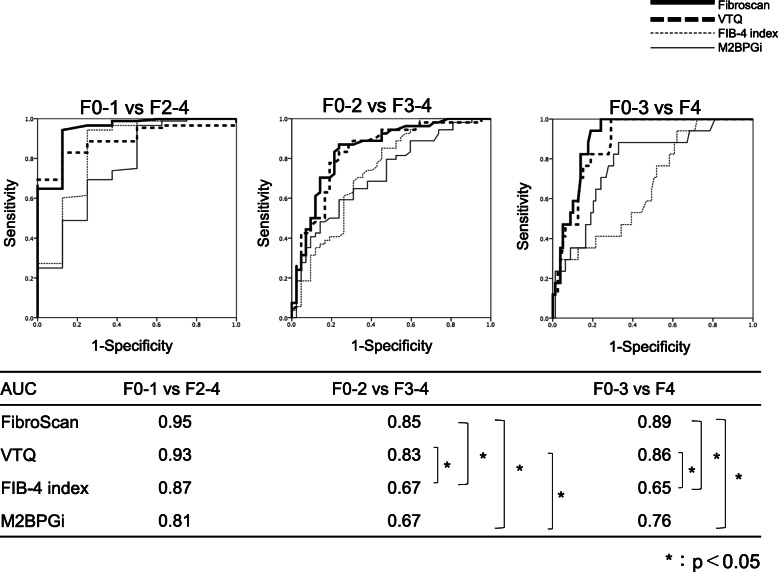
Comparison of ROC curves representing four different methods for the diagnosis of liver fibrosis using liver specimens as the reference. F0–1 versus F2–4, F0–2 versus F3–4, F0–3 versus F4. ROC curves: Receiver operating characteristic curves

The sensitivity, specificity, and cut-off values were compared among the four diagnostic methods. The ROC curves for each method are shown in (Fig. 4). The areas under the ROC curve (AUC) for diagnosis of fibrosis stage F2 or greater were as follows: 0.95 for FibroScan, 0.93 for VTQ, 0.87 for the FIB-4 index, and 0.81 for the M2BPGi level. The respective values for diagnosis of grade F3 or greater were 0.85, 0.83, 0.67, and 0.67, and those for diagnosis of F4 were 0.89, 0.86, 0.65, and 0.76 (Fig. 4). In the ROC comparison, there was a significant difference in “VTQ vs FIB-4 index”, “FibroScan vs FIB-4 index”, “FibroScan vs M2BPGi”, “VTQ vs M2BPGi”in the F0–2 vs F3–4 group, “VTQ vs FIB-4 index”, “FibroScan vs FIB-4 index”, “FibroScan vs M2BPGi” in the F0–3 vs F4 group (Fig. 4). The cutoff values for each test for a diagnosis of grade F2 or greater were as follows: FibroScan, 5.6; VTQ, 1.26; FIB-4 index, 1.74; M2BPGi, 1.63. The respective values for a diagnosis of F3 or greater were 9.8, 1.78, 3.20, and 2.15, and those for a diagnosis of F4 were 16.0, 1.94, 4.56, and 2.70 (Table 3).

**Table 3:** Sensitivity, specificity, and diagnostic accuracy of cut-off and area under the curve values for evaluating liver stiffness in all patients with liver tumors and hepatitis C viral infection

HCC *n* = 94 + control *n* = 14		Sensitivity (%)	Specificity (%)	PPV (%)	NPV (%)	cut off	AUC	AUC (*p* value)			
								vs FibroScan	vs VTQ	vs FIB-4 index	vs M2BPGi
F0–1 (*n* = 16) vs F2–4 (*n* = 92)	FibroScan	95	86	90	73	5.6	0.95	Ref	0.602	0.110	0.211
	VTQ	87	86	95	54	1.26	0.93	0.602	Ref	0.233	0.361
	FIB-4 index	98	72	93	83	1.74	0.87	0.110	0.233	Ref	0.822
	M2BPGi	74	71	96	76	1.63	0.81	0.211	0.361	0.822	Ref
F0–2 (*n* = 52) vs F3–4 (*n* = 56)	FibroScan	88	74	84	83	9.8	0.85	Ref	0.649	0.002	0.001
	VTQ	81	81	87	76	1.78	0.83	0.649	Ref	0.010	0.002
	FIB-4 index	73	52	69	65	3.20	0.67	0.002	0.010	Ref	0.471
	M2BPGi	66	59	70	55	2.15	0.67	0.001	0.002	0.471	Ref
F0–3 (*n* = 91) vs F4 (*n* = 17)	FibroScan	83	82	63	97	16.0	0.89	Ref	0.453	0.002	0.029
	VTQ	92	70	65	98	1.94	0.86	0.453	Ref	0.045	0.087
	FIB-4 index	58	55	67	88	4.56	0.65	0.002	0.045	Ref	0.215
	M2BPGi	75	62	66	93	2.70	0.76	0.029	0.087	0.215	Ref

*VTQ* Virtual-Touch tissue quantification, *M2BPGi* Mac-2 binding protein glycosylation isomer, *AUC* area under the curve, *PPV* positive predictive value, *NPV* negative predictive value

#### Non-SVR group

**Fig. 5:**
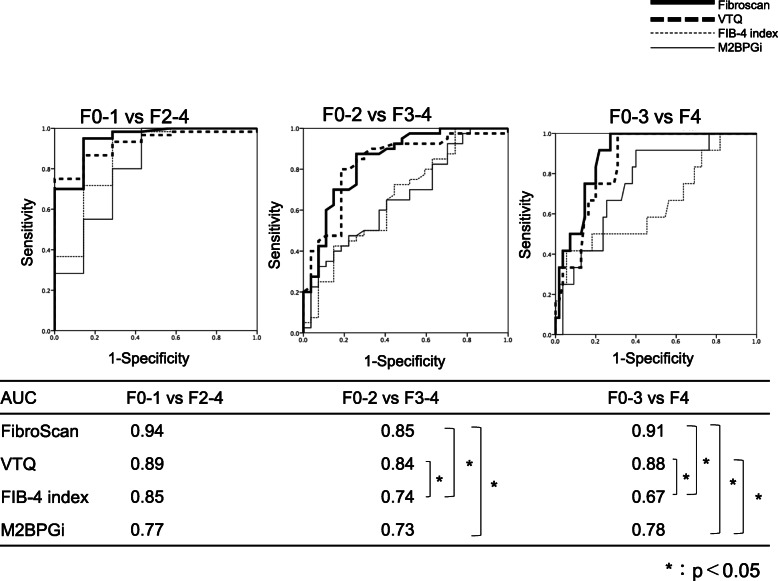
ROC analyses of different modalities for the diagnosis of liver fibrosis in the non-SVR group using liver specimens as the reference. F0–1 versus F2–4, F0–2 versus F3–4, F0–3 versus F4. ROC curves: Receiver operating characteristic curves

The results for the non-SVR group are shown in (Fig. 5). The fibrosis stage of the 14 control patients were defined as F0–1 in the analysis. The AUC for diagnosis of fibrosis stage F2 or greater were as follows: 0.94 for FibroScan, 0.89 for VTQ, 0.85 for the FIB-4 index, and 0.77 for the M2BPGi level. The respective values for a diagnosis of grade F3 or greater were 0.85, 0.84, 0.74, and 0.73, and those for a diagnosis of F4 were 0.91, 0.88, 0.67, and 0.78. In the ROC comparison, there were significant differences in “VTQ vs FIB-4 index”, “FibroScan vs FIB-4 index”, “FibroScan vs M2BPGi” in the F0–2 vs F3–4 group, “VTQ vs FIB-4 index”, “FibroScan vs FIB-4 index”, “FibroScan vs M2BPGi”, “VTQ vs M2BPGi” in the F0–3 vs F4 group. The cutoff values for each test for a diagnosis of grade F2 or greater were as follows: FibroScan, 6.2; VTQ, 1.27; FIB-4 index, 1.74; M2BPGi, 1.40. The respective values for a diagnosis of F3 or greater were 8.9, 1.46, 2.91, and 1.76, and those for a diagnosis of F4 were 15.0, 1.94, 3.25, and 2.70. (Table 4).

**Table 4:** Sensitivity, specificity, and diagnostic accuracy of cut-off and area under the curve values for evaluating liver stiffness in non-SVR patients with liver tumors and hepatitis C viral infection

non-SVR *n* = 64 + control *n* = 14		Sensitivity (%)	Specificity (%)	PPV (%)	NPV (%)	cut off	AUC	AUC (*p* value)			
								vs FibroScan	vs VTQ	vs FIB-4 index	vs M2BPGi
F0–1 (*n* = 15) vs F2–4 (*n* = 63)	FibroScan	93	82	93	81	6.2	0.94	Ref	0.226	0.110	0.211
	VTQ	82	88	98	88	1.27	0.89	0.226	Ref	0.273	0.361
	FIB-4 index	91	69	95	63	1.74	0.85	0.110	0.273	Ref	0.822
	M2BPGi	70	63	83	81	1.40	0.77	0.211	0.361	0.822	Ref
F0–2 (*n* = 36) vs F3–4 (*n* = 42)	FibroScan	91	72	88	71	8.9	0.85	Ref	0.694	0.020	0.003
	VTQ	87	82	88	71	1.46	0.84	0.694	Ref	0.049	0.304
	FIB-4 index	78	62	79	62	2.91	0.74	0.020	0.049	Ref	0.304
	M2BPGi	70	62	70	69	1.76	0.73	0.003	0.304	0.304	Ref
F0–3 (*n* = 65) vs F4 (*n* = 13)	FibroScan	94	80	79	79	15.0	0.91	Ref	0.593	0.003	0.026
	VTQ	94	75	75	75	1.94	0.88	0.593	Ref	0.006	0.049
	FIB-4 index	67	57	55	55	3.25	0.67	0.003	0.006	Ref	0.190
	M2BPGi	76	69	74	74	2.70	0.78	0.026	0.049	0.190	Ref

*VTQ* Virtual-Touch tissue quantification, *M2BPGi* Mac-2 binding protein glycosylation isomer, *AUC* area under the curve, *PPV* positive predictive value, *NPV* negative predictive value

#### SVR group

The results for the SVR group are shown in (Figure 1S). The fibrosis stages of the 14 control patients were defined as F0–1 in the analysis. The AUC for a diagnosis of fibrosis stage F2 or greater were as follows: 0.98 for FibroScan, 0.78 for VTQ, 0.78 for the FIB-4 index, and 0.63 for the M2BPGi level. The respective values for a diagnosis of grade F3 or greater were 0.91, 0.89, 0.89, and 0.77, and those for a diagnosis of F4 were 0.94, 0.94, 0.77, and 0.77 (Figure 1S). In the ROC comparison, there were significant differences in “FibroScan vs VTQ”, “FibroScan vs FIB-4 index”, “FibroScan vs M2BPGi” in the F0–1 vs F2–4 group, “FibroScan vs FIB-4 index” in the F0–3 vs F4 group. The cutoff values for each test for a diagnosis of grade F2 or greater were as follows: FibroScan, 6.2; VTQ, 1.27; FIB-4 index, 1.90; M2BPGi, 1.00. The respective values for a diagnosis of F3 or greater were 9.6, 1.56, 2.70, and 1.50, and those for a diagnosis of F4 were 21.5, 2.63, 2.82, and 2.56. (Table 1S).

## Discussion

Many reports have compared the METAVIR scores of FibroScan and VTQ with liver biopsy, and both have been reported to be highly reliable [12, 15–18]. As mentioned previously, however, there are cases in which liver biopsy cannot provide accurate or objective results. Only a few reports have compared liver resection specimens with FibroScan, VTQ, and M2BPGi [19, 25, 27, 28]. In this study, we compared four different methods used to evaluate liver resection specimens: FibroScan, VTQ, the FIB-4 index, and M2BPGi level. In the comparisons of METAVIR scores with each examination method, the correlation coefficients between the METAVIR score and FibroScan and between METAVIR and VTQ were very similar, and these two methods seemed to predict the degree of hepatic fibrosis better than the other two methods. In the ROC curve analyses, both FibroScan and VTQ exhibited AUC values greater than 0.8 for each METAVIR fibrosis stage, indicating better sensitivity and specificity. These results suggest that FibroScan and VTQ accurately reflect the hepatic fibrosis stage in liver resection specimens.

Ragazzo et al. compared FibroScan, VTQ, the enhanced liver fibrosis test, the aspartate aminotransferase to platelet ratio index, and the FIB-4 index with liver biopsy results for evaluation of fibrosis [12]. In that study, FibroScan and VTQ remained the most effective methods for evaluating all degrees of fibrosis. The accuracy of all methodologies was best at F4 [12]. However, the authors did not examine SVR and non-SVR status. Vallet-Pichard et al. compared the FIB-4 index with the Fibro test and METAVIR scores, respectively, [10] and found that FIB-4 index was associated with Fibro test and METAVIR scores. However, the study also did not distinguish between SVR and non-SVR. Therefore, these studies may be influenced by SVR status.

Nagata et al. and Chen et al. reported that FIB4-Index and M2BPGi levels are significantly decreased regardless of degree of fibrosis in SVR patients [20]. In addition, Bachofner et al. reported that FIB-4 index levels are also decreased in SVR patients [24, 25]. SVR status of patients in this study is shown in Table 2. The AST, ALT and HCC stages of SVR patients tended to be lower than those of non-SVR patients. In this study, the AUC of FIB-4 index and M2BPGi appeared to be low, which seemed to be due to the inclusion of SVR patients. Taking these effects into consideration, a similar study was conducted focusing on SVR and non-SVR patients separately. Although the values were different, the benefits of FibroScan and VTQ did not change in non-SVR patients. Superiority of blood data was observed in SVR patients compared to other studies. However, since the number of SVR patients is small, it would be desirable to consider increasing the number.

To summarize the comparisons between our results and those of past reports, the diagnosis of hepatic fibrosis using ultrasound has a slightly different value in VTQ, but no significant difference in the AUC was observed. However, when using blood-based parameters (i.e., the FIB-4 index and M2BPGi level) to diagnose fibrosis, the AUCs were lower in this study than in previous reports. The presence of HCC, HCV infection, inflammation, and differences by gender may have influenced the results. Sato et al. reported differences in blood test data depending on the presence of HCC. It has been reported that M2BPGi (≥2.8 COI) tends to increase as HCC develops, and the FIB-4 index (≥3.7) is high when HCC is present [24]. It may be important that M2BPGi and the FIB-4 index were calculated in HCC-free patients.

Compared with blood-based parameters, AUC scores associated with ultrasound-based parameters were higher than those associated with blood-based parameters in almost all previous reports. Regarding these differences, ultrasonic parameters can be used to evaluate the liver specifically, while blood-based parameters such as the FIB-4 index and M2BPGi level, are influenced by factors outside the liver. As noted above, the FIB-4 index and M2BPGi level yielded relatively poor results in comparison with previous studies [10–13]. According to the ROC analyses, the sensitivity and specificity of the FIB-4 index and M2BPGi level for diagnosing fibrosis decreased as liver fibrosis progressed. However, the detection of fibrosis using blood-based parameters was equivalent to that using FibroScan and VTQ for mild fibrosis stages.

There are several limitations in this study. The number of patients included in the study was small. Especially, there were only two patients of F0–1 cases with HCC. Therefore, patients with liver metastasis were included in all studies as a control in F0–1 cases. And the study population was restricted to HCC patients; blood-based parameters may be altered by the presence of HCC, which might affect the accuracy of the results. Future studies including other patient groups will be necessary for replication. In addition, there were 32 patients for whom the measurement site and resected specimen differed. Because there is a possibility of measurement error resulting from this, it would be preferable for the excision site and the measurement part to be identical. There was also some discrepancy in the results of two ultrasonic elastographies. Although it is reasonable that the presence of HCC and gender-related differences might affect the results, these effects could not be investigated in this study. In addition, there were 11 patients in the study cohort who were treated by transcatheter arterial chemoembolization as neoadjuvant treatment prior to surgical resection of the tumor. These treatments might have resulted in hepatocyte necrosis or inflammation, which can confound liver stiffness or FIB-4 index and M2BPGi measurements.

## Conclusion

In conclusion, FibroScan and VTQ were associated with the diagnosis of liver fibrosis using hepatectomy specimens.

## Supplementary information

**Additional file 1: Supplementary data Figure 1.** ROC analyses of different modalities for the diagnosis of various stages of liver fibrosis in the SVR group using liver specimens as the reference. F0–1 versus F2–4, F0–2 versus F3–4, F0–3 versus F4. ROC curves: Receiver operating characteristic curves

**Additional file 2: Supplementary Table 1.** Sensitivity, specificity, and diagnostic accuracy of cut-off and area under the curve values for evaluating liver stiffness in SVR patients with liver tumors and hepatitis C viral infection

## Data Availability

The datasets used and analyzed during the current study are available from the corresponding author on reasonable request.

## Abbreviations

HCC: Hepatocellular carcinoma
VTQ: Virtual-Touch tissue quantification
FIB-4 index: Fibrosis index based on four factors
M2BPGi: Mac-2 binding protein glycosylation isomer
HCV: Hepatitis C virus
ROC: Receiver operating characteristic
AUC: The area under the curve
