# Supplementary material for: Liver fibrosis assessments using FibroScan, virtual-touch tissue quantification, the FIB-4 index, and mac-2 binding protein glycosylation isomer levels compared with pathological findings of liver resection specimens in patients with hepatitis C infection
Source: BMC Gastroenterol. 2020 Sep 25;20:314. doi: 10.1186/s12876-020-01459-w (PMC7519502; doi:10.1186/s12876-020-01459-w)
Supplement: Supplementary file 1 — Additional file 1: Supplementary data Figure 1. ROC analyses of different modalities for the diagnosis of various stages of liver fibrosis in the SVR group using liver specimens as the reference. F0–1 versus F2–4, F0–2 versus F3–4, F0–3 versus F4. ROC curves: Receiver operating characteristic curves [file 12876_2020_1459_MOESM1_ESM.docx]

 Figure 5 supplementary data. ROC analyses of different modalities for the diagnosis of various stages of liver fibrosis in the SVR group using liver specimens as the reference. F0–1 versus F2–4, F0–2 versus F3–4, F0–3 versus F4. ROC curves : Receiver operating characteristic curves
