# Supplementary material for: Liver fibrosis assessments using FibroScan, virtual-touch tissue quantification, the FIB-4 index, and mac-2 binding protein glycosylation isomer levels compared with pathological findings of liver resection specimens in patients with hepatitis C infection
Source: BMC Gastroenterol. 2020 Sep 25;20:314. doi: 10.1186/s12876-020-01459-w (PMC7519502; doi:10.1186/s12876-020-01459-w)
Supplement: Supplementary file 2 — Additional file 2: Supplementary Table 1. Sensitivity, specificity, and diagnostic accuracy of cut-off and area under the curve values for evaluating liver stiffness in SVR patients with liver tumors and hepatitis C viral infection [file 12876_2020_1459_MOESM2_ESM.docx]

Table 4 Supplementary. Sensitivity, specificity, and diagnostic accuracy of cut-off and area under the curve values for evaluating liver stiffness in SVR patients with liver tumors and hepatitis C viral infection

| SVR n=30  +  control n=14 | | Sensitivity  (%) | Specificity  (%) | PPV  (%) | NPV  (%) | cut off | AUC | AUC (p value) | | | |
| --- | --- | --- | --- | --- | --- | --- | --- | --- | --- | --- | --- |
|  |  |  |  |  |  |  |  | vs FibroScan | vs  VTQ | vs FIB-4 index | vs M2BPGi |
| F0-1 (n=15)  vs  F2-4 (n=29) | FibroScan | 96 | 88 | 97 | 87 | 6.2 | 0.98 | Ref | 0.04 | 0.02 | 0.02 |
|  | VTQ | 74 | 75 | 76 | 87 | 1.27 | 0.78 | 0.04 | Ref | 0.58 | 0.17 |
|  | FIB-4 index | 78 | 75 | 83 | 73 | 1.90 | 0.78 | 0.02 | 0.58 | Ref | 0.23 |
|  | M2BPGi | 59 | 50 | 62 | 73 | 1.00 | 0.63 | 0.02 | 0.17 | 0.23 | Ref |
| F0-2 (n=30)  vs  F3-4 (n=14) | FibroScan | 85 | 82 | 86 | 90 | 9.6 | 0.91 | Ref | 0.83 | 0.67 | 0.07 |
|  | VTQ | 85 | 82 | 79 | 87 | 1.56 | 0.89 | 0.83 | Ref | 0.83 | 0.18 |
|  | FIB-4 index | 85 | 87 | 93 | 80 | 2.70 | 0.89 | 0.67 | 0.83 | Ref | 0.11 |
|  | M2BPGi | 69 | 69 | 71 | 80 | 1.50 | 0.77 | 0.07 | 0.18 | 0.11 | Ref |
| F0-3 (n=40)  vs  F4 (n=4) | FibroScan | 75 | 99 | 75 | 93 | 21.5 | 0.94 | Ref | 0.89 | 0.04 | 0.29 |
|  | VTQ | 75 | 99 | 75 | 93 | 2.63 | 0.94 | 0.89 | Ref | 0.06 | 0.21 |
|  | FIB-4 index | 75 | 68 | 75 | 68 | 2.82 | 0.77 | 0.04 | 0.06 | Ref | 0.90 |
|  | M2BPGi | 75 | 77 | 75 | 85 | 2.56 | 0.77 | 0.29 | 0.21 | 0.90 | Ref |

VTQ: Virtual-Touch tissue quantification, M2BPGi: Mac-2 binding protein glycosylation isomer, AUC: area under the curve, PPV: positive predictive value, NPV: negative predictive value.
